# Safety and efficacy of a two-screw cephalomedullary nail for intertrochanteric femur fracture fixation: a retrospective case series in 264 patients

**DOI:** 10.1186/s13037-018-0177-x

**Published:** 2018-11-06

**Authors:** Boris A. Zelle, Antonio J. Webb, Christopher Matson, Michael Morwood, Khang H. Dang, Samuel S. Ornell, Gabrielle Gostigian, Cody M. Ramirez, Hassan Mir

**Affiliations:** 1 Department of Orthopaedics, UT Health San Antonio, 7703 Floyd Curl Dr, MC-7774, San Antonio, TX 78229 USA; 2 Department of Orthopaedics, University of South Florida, Tampa, FL 33606 USA; 3 Department of Orthopaedics, Florida Orthopedic Institute, Tampa, FL 33606 USA

**Keywords:** Intertrochanteric fracture, Cephalomedullary nail, Mechanical failure, Safety

## Abstract

**Introduction:**

Recent advances have led to the design of a new cephalomedullary nail, which aims to decrease the risk of failures in patients with intertrochanteric hip fractures by allowing for insertion of two interdigitating screws into the head segment. The goal of this study is to evaluate the safety and efficacy of this two-screw cephalomedullary nailing system.

**Patients/participants:**

Patients 18 years of age and older who underwent intramedullary nailing of their intertrochanteric femoral fracture using the InterTAN nailing system (Smith and Nephew, Memphis, TN) from 2012 to 2016 were included in this retrospective study which was performed at two urban certified level-1 trauma centers and one urban certified level-3 trauma center. The study data was collected through a retrospective chart review and review of the existing radiographic studies. Primary outcome measure was mechanical hardware failure and screw cutout. Secondary outcome measures included nonunion, malunion, medical and surgical complications.

**Results:**

A total of 264 patients were included in this analysis. Two patients (0.75%) were found to have a screw cut out requiring revision surgery. Two other revision surgeries were performed for malrotation (*n* = 1) and malunion (*n* = 1). Other implant-related complications occurred in 19 cases (7.9%), which included broken distal screws (*n* = 9), distal screw loosening (*n* = 8), and loose lag screws (*n* = 2). There was a total of 10 (3.8%) surgical wound complications, including four deep and six superficial infections.

**Discussion:**

This modified cephalomedullary nail is a reliable, safe, and effective implant for management of intertrochanteric hip fractures. Surgical treatment of patients with intertrochanteric hip fractures can be performed in a safe fashion using this implant.

## Introduction

Intertrochanteric fractures account for a vast majority of hip fractures in the elderly population [1–6]. Several surgical options, such as the sliding hip compression screw, intramedullary nail, and arthroplasty, exist for the management of these fractures [7]. Due to relative ease of use and favorable clinical outcomes, cephalomedullary nailing has become one of the most common means of hip fixation in the United States [1, 8]. However, complications do arise after implantation of these nails, including mechanical failure, screw cut out, varus collapse, shortening of the femoral neck, and peri-implant femoral shaft fractures around the distal tip of the implant [9]. Recently, a new two-screw cephalomedullary nail with integrated interlocking lag and compression screws was designed to minimize these complications and to improve patient safety in surgery.

In contrast to many available chephalomedullary nailing systems, this modified cephalomedullary nail provides a fixation construct with two integrated interlocking lag and compression screws and a trapezoidal nail profile designed to optimize stability [10]. Thus, the insertion of a lag screw combined with an interdigitated compression screws may potentially minimize the risk of screw cut out from the head segment by providing immediate intraoperative linear compression, improved rotational stability, and increased bony purchase within the femoral head. The interdigitating screw insertion further allows for minimizing the risk of the reported Z-effect, which has been described as lateral and medial migration of the superior and inferior screws respectively [11].

The benefit for patient safety of this two-screw cephalomedullary nailing system requires further clinical investigations. The principal goal of this study was to examine the mechanical failure rates and to determine the safety and efficacy of this cephalomedullary nailing system. We hypothesize that the mechanical failure rates associated with this nailing system compare favorably with the results of other cephalomedullary nailing systems reported in the literature.

## Materials and methods

This was a retrospective study that was performed at two urban certified level-1 trauma centers and one urban certified level-3 trauma center. The study data was collected through a retrospective chart review and review of the existing radiographic studies. Patients were identified through the coding database of our institution. Approval of the study protocol was obtained from each Institutional Review Board (IRB) at the respective institutions.

Patients 18 years of age and older who underwent nail fixation of their acute intertrochanteric femoral fracture using the InterTAN cephalomedullary nail (Smith and Nephew, Memphis, TN) between 2012 and 2016 were included in this investigation. Fractures were classified using the OTA/AO fracture classification [12]. Intertrochanteric fractures treated with sliding hip compression screws, arthroplasty, or other nailing systems were excluded from this study. Patients with pathologic fractures from neoplastic disease or femoral head/neck fractures were also excluded from this study.

**Fig. 1:**
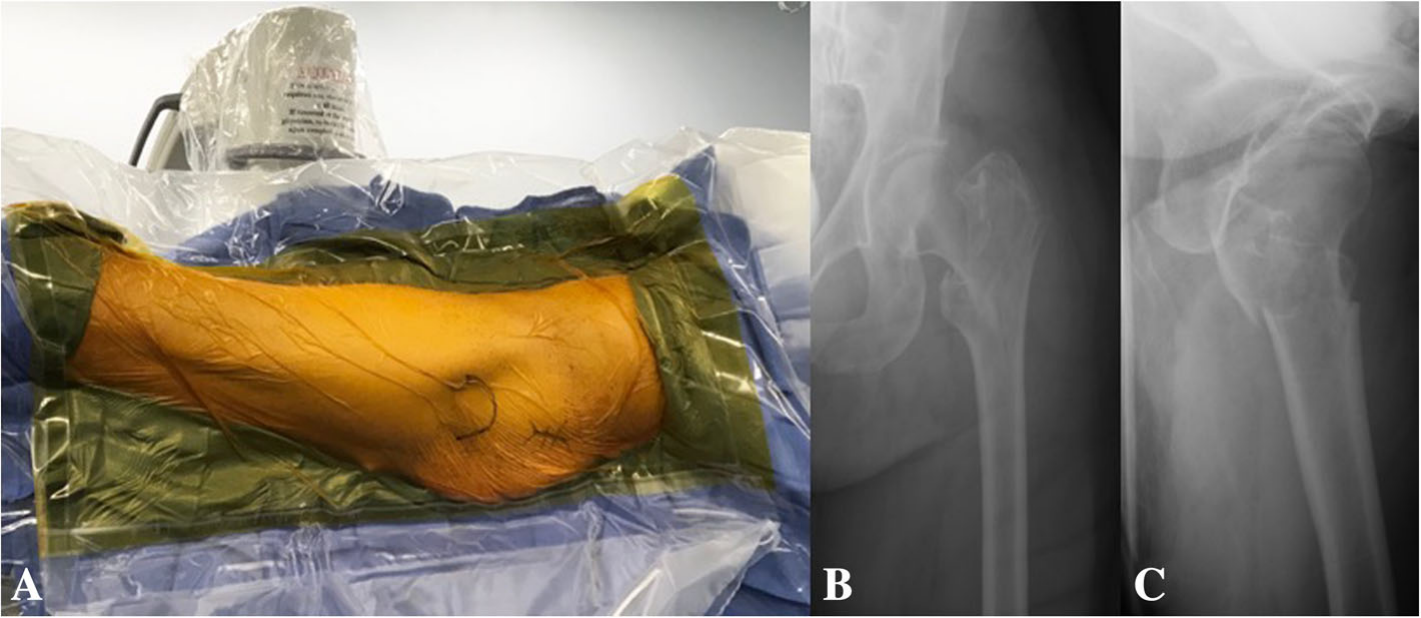
**a**-**c**. Unstable intertrochanteric femur fracture with lateral wall involvement (1**a**-**b**). Sterile preparation of patient on fracture table with incision marked approximately three fingerbreadths above the greater trochanter (1**c**)

**Fig. 2:**
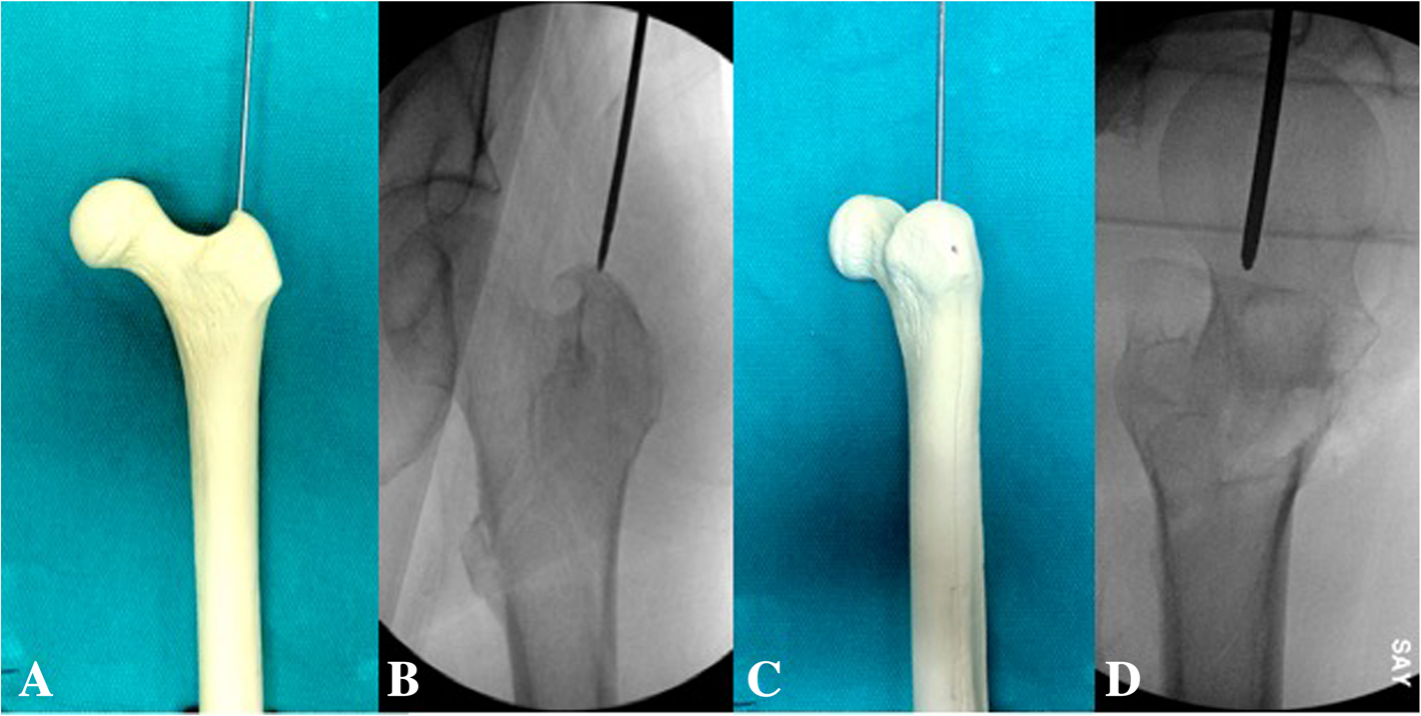
**a-d**. Starting point at greater trochanter as demonstrated on AP (2**a**-**b**) and lateral view (2**c**-**d**)

**Fig. 3:**
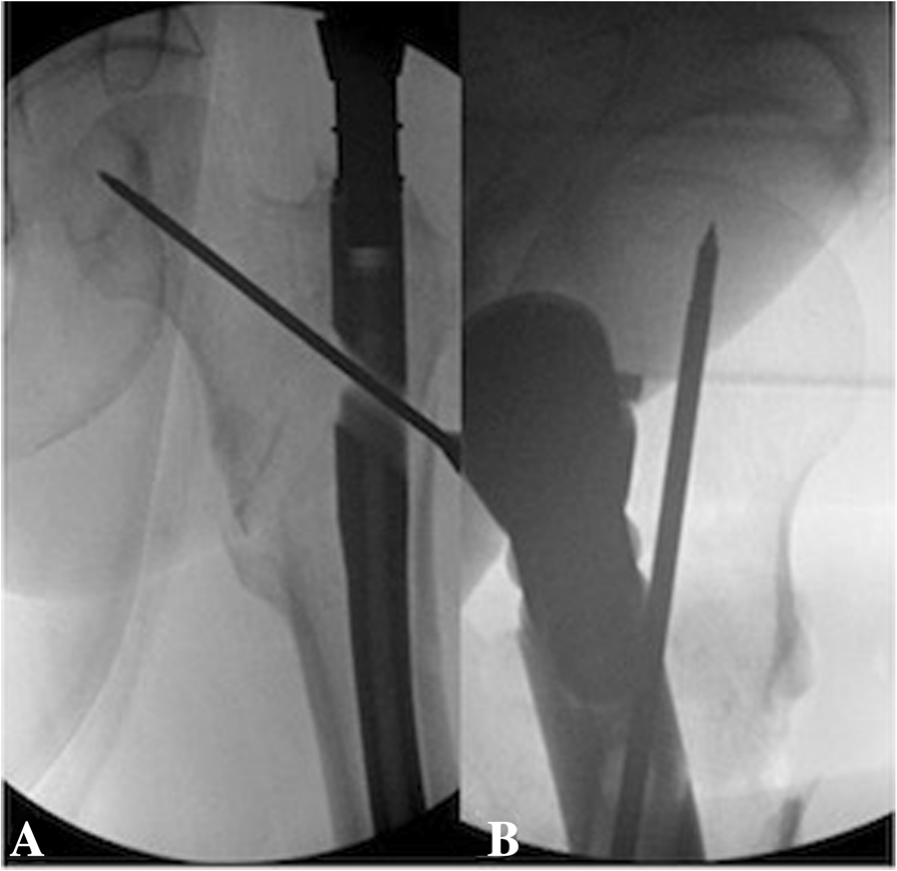
**a-b**. Pin position for lag screw placement on AP (3**a**) and lateral view (3**b**)

**Fig. 4:**
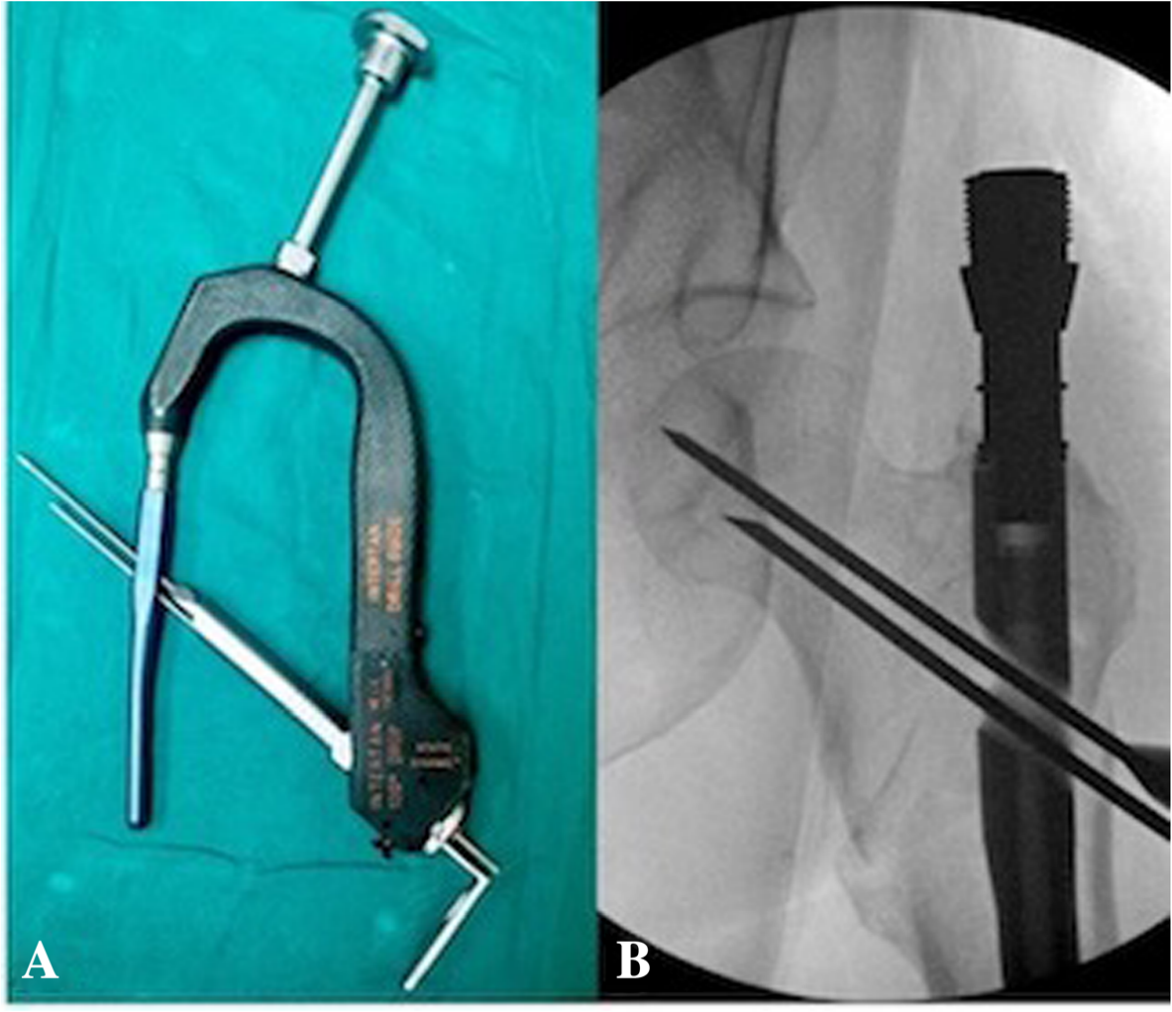
**a-b**. Placement of anti-rotation bar

**Fig. 5:**
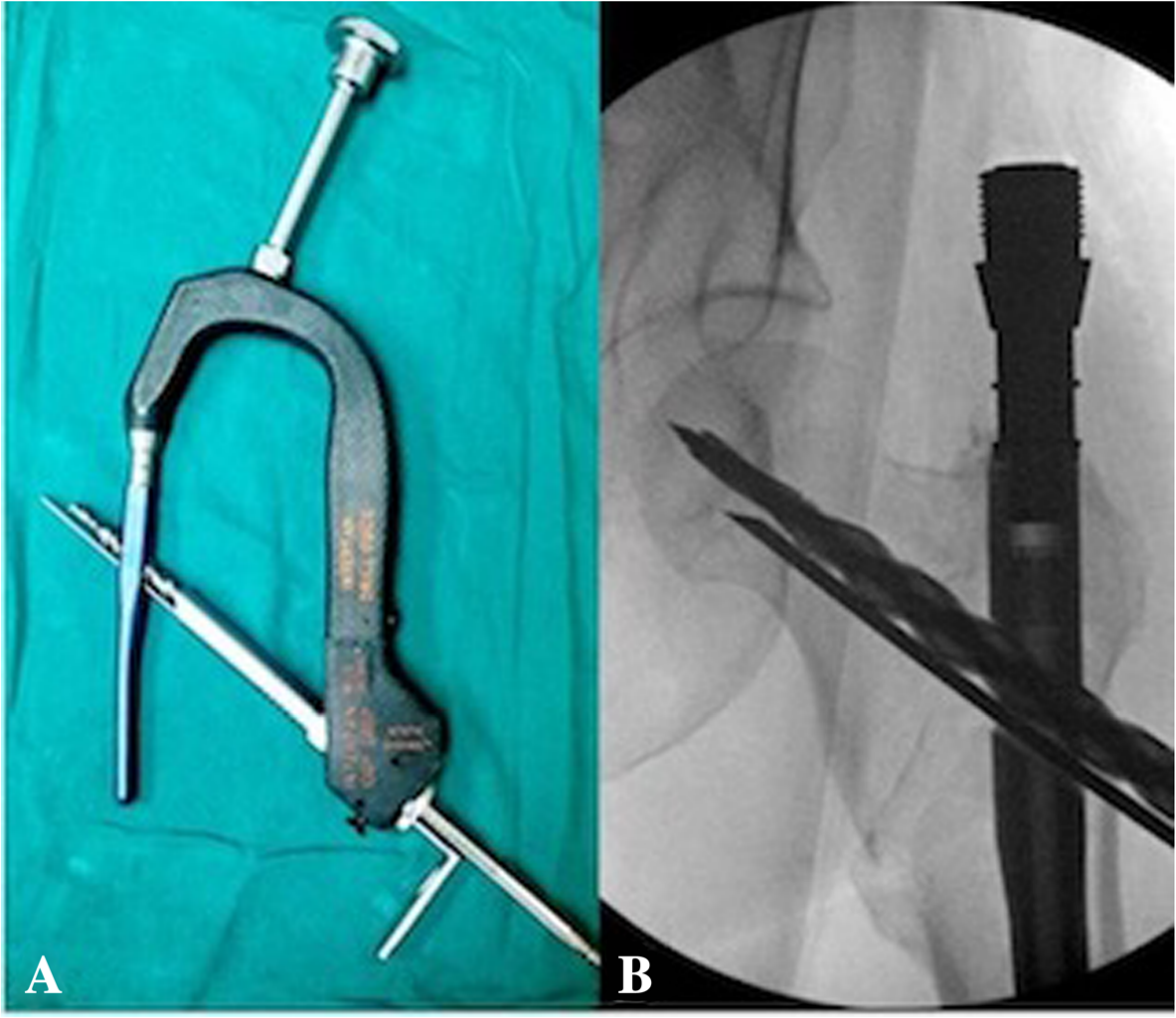
**a-b**. Drilling for the lag screw

**Fig. 6:**
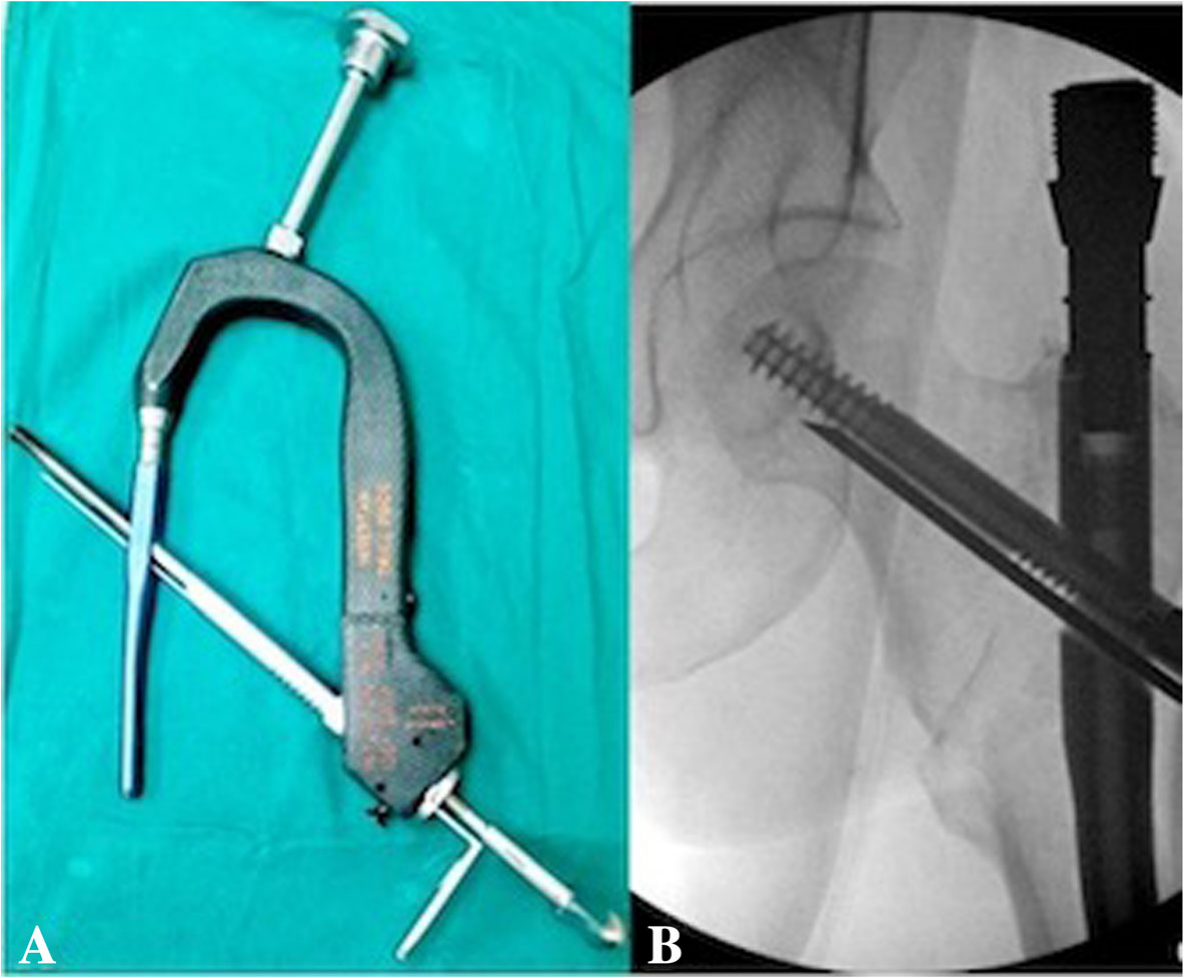
**a-b**. Insertion of the lag screw with the anti-rotation bar in place

**Fig. 7:**
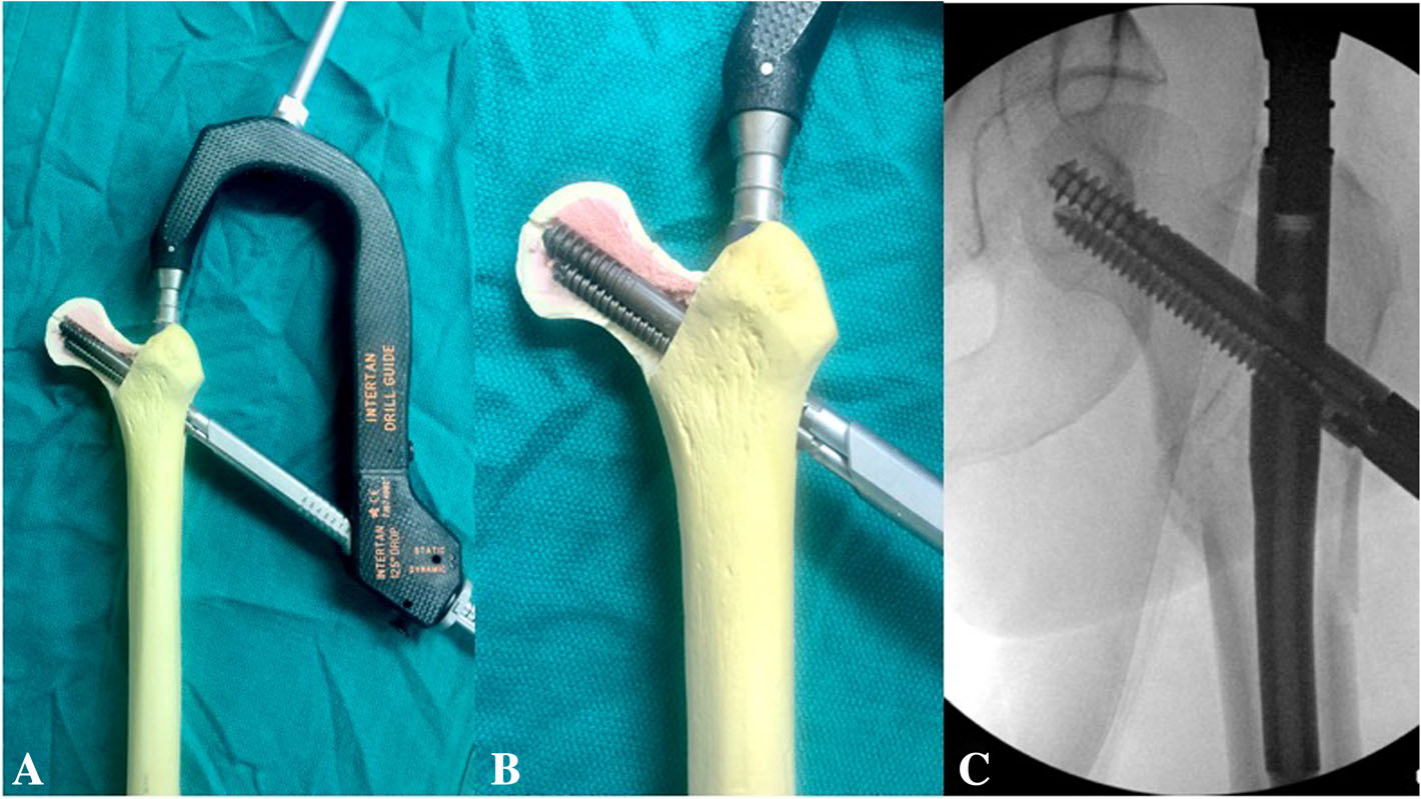
**a-c**. Placement of compression screw interdigitating with the lag screw

**Fig. 8:**
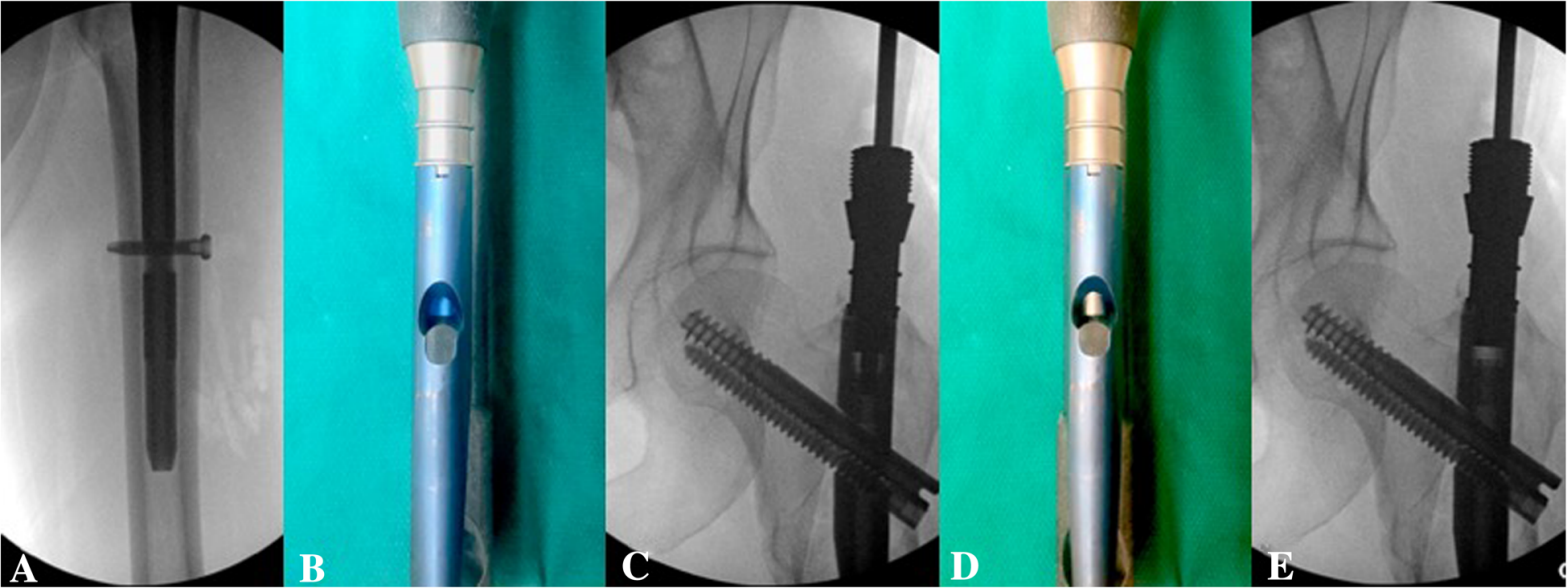
**a-e**. Additional stability by distal interlocking screw (8**a**). Side view of the nail and AP fluoroscopic picture before (8**b**-**c**) and after (8**d**-**e**) tightening down the set screw

**Fig. 9:**
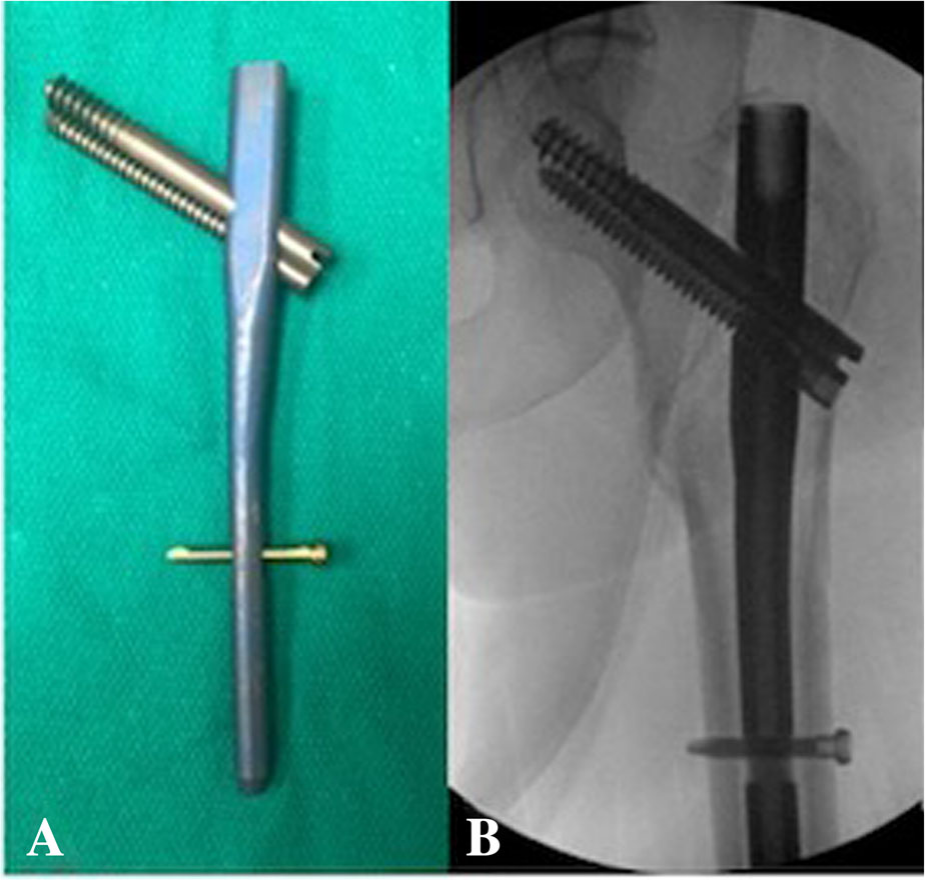
**a-b**. Final construct (9**a**) with corresponding AP fluoroscopic picture (9**b**)

**Fig. 10:**
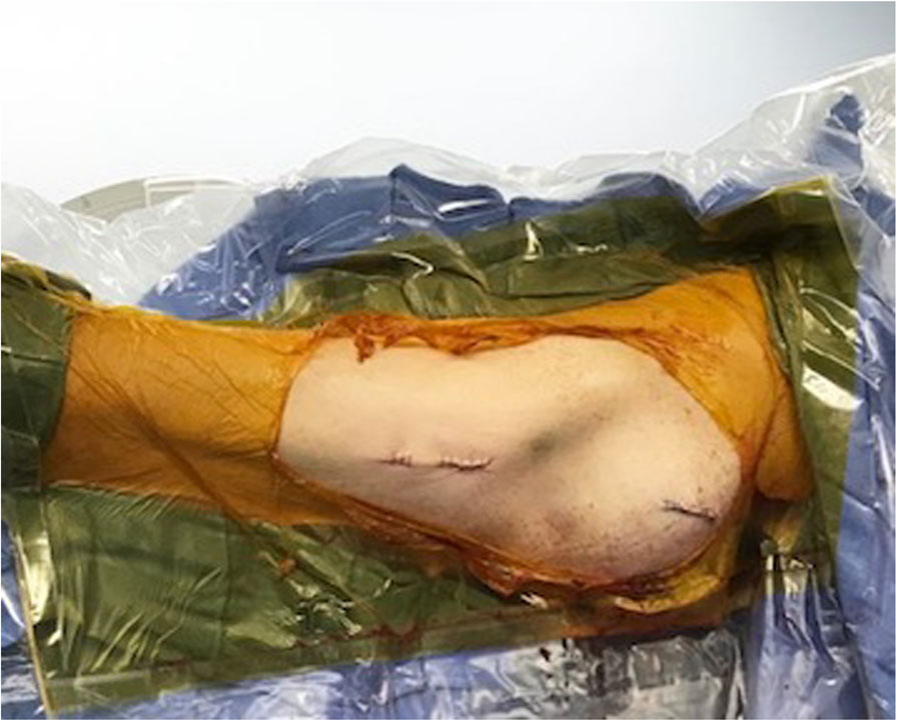
Final closure after minimal-invasive procedure through three relatively small incisions

**Fig. 11:**
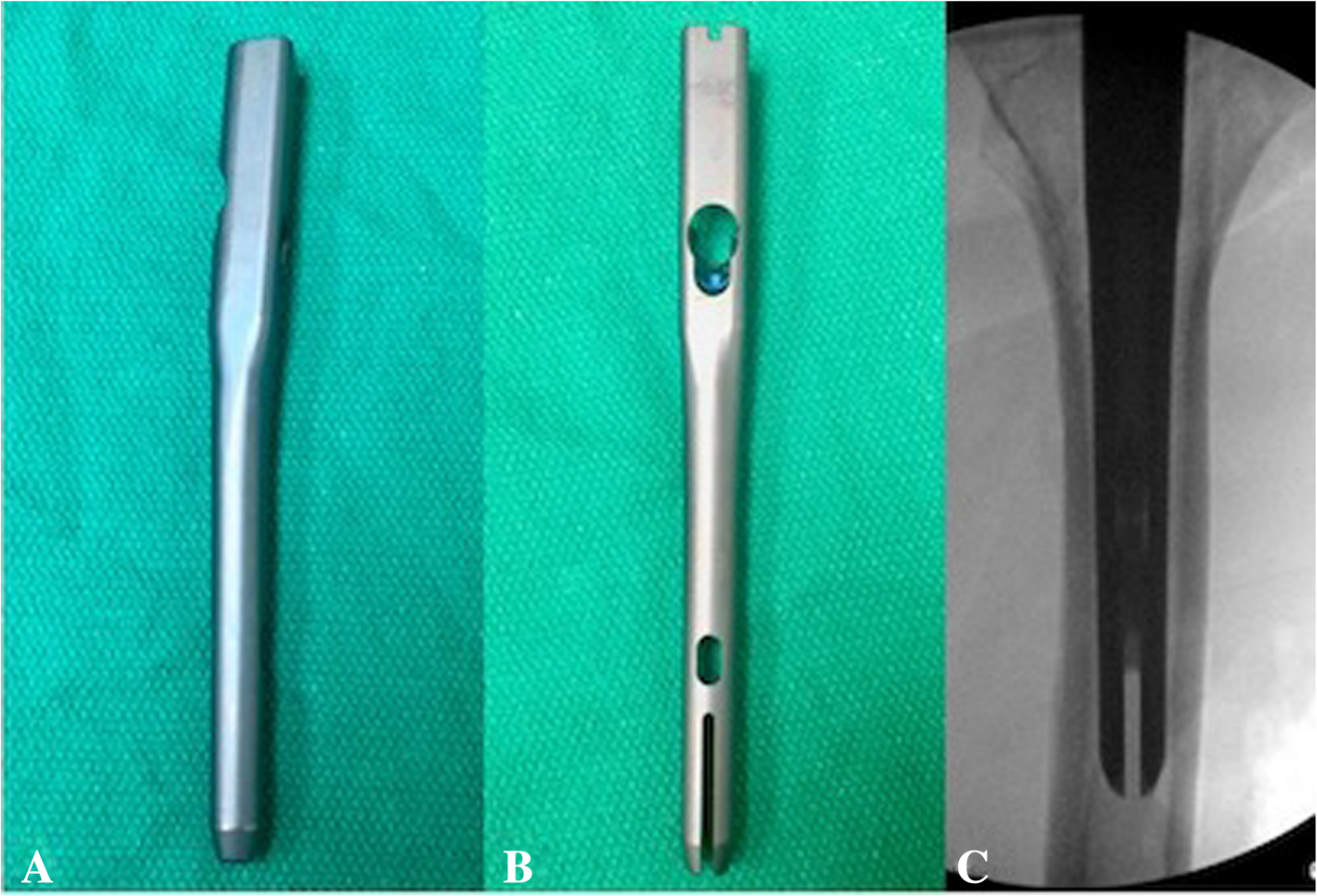
**a-c**. InterTAN nail construct front view (11**a**) and lateral view (11**b**) showing trapezoidal proximal nail profile. Clothespin distal tip seen on lateral view of the nail (11**b**) lateral fluoroscopic image (11**c**)

All patients included in this study underwent cephalomedullary nailing using the InterTAN. The surgical technique was according to widely established recommendations as described in the literature and according to manufacturer guidelines [10, 13–17]. In brief, the patient is placed on a standard fracture table to allow for application of traction and appropriate fracture reduction (Fig. 1). An approximately 3–4 cm surgical incision is made approximately 3 fingerbreadths proximal to the greater trochanter. The fascia is incised and a 3.2 mm guide pin is placed on the appropriate entry point which is located on the medial face of the greater trochanter on the anteroposterior (AP) fluoroscopic view and in-line with the femoral canal on the lateral fluoroscopic view (Fig. 2). Following appropriate guide pin placement, the canal is opened with an entry reamer and a ball-tipped guide-wire is placed into the femoral canal. We recommend reaming the femoral canal for appropriate preparation of the nail insertion. Once the diameter and length of the nail has been determined, the nail is assembled with the drill guide, and advanced into the femoral canal. Following nail insertion, the appropriate depth and alignment is confirmed on both AP and lateral fluoroscopic views of the hip. The lag screw position is planned with a 3.2 mm guide pin that is inserted through the aiming jig. The lag screw position follows general guidelines with an appropriate tip-apex distance and a center/center position on both the AP and lateral fluoroscopic view (Fig. 3). Then a 7- mm drill is inserted through the aiming jig to drill for the compression screw just below the lag screw. Afterwards, an anti-rotation bar is placed into the drill hole for the compression screw in order to avoid spinning of the head segment during insertion of the lag screw (Fig. 4). The lag screw is drilled over the guide pin (Fig. 5) and the lag screw is inserted with the anti-rotation bar in place (Fig. 6). The compression screw is placed providing linear compression across the fracture site and additional stability by interdigitation with the lag screw (Fig. 7). It is recommended to release the traction during insertion of the compression screw in order to allow for appropriate linear compression. Additional stability can be achieved by insertion of a distal interlocking screw and by tightening down the proximal set screw (Fig. 8). Appropriate fracture reduction and implant position is confirmed on final AP and lateral fluoroscopic views (Fig. 9). The wounds are closed in a standard fashion. The procedure can usually be performed in a minimal invasive fashion through three relatively small incisions (Fig. 10). Additional safety features of this nailing system include a trapezoidal shape in the proximal portion providing rotational stability, press-fit in the metaphyseal region, and distribution of tensile forces. In addition, the clothespin distal tip is less rigid to decrease the stress riser, potentially reducing the incidence of periprosthetic fractures and anterior thigh pain (Fig. 11).

Patients were considered as incomplete follow-up if clinical and radiographic outcome data was not available for a minimum of 12 weeks after surgery. Technical data on the fixation construct and the mechanical failures were collected from the postoperative radiographs. Radiographic data recorded included the nail size (short vs. long), neck shaft angle, tip apex distance, and the number of distal locking screws.

The primary outcome measures were mechanical hardware failure and proximal screw cutout. Secondary outcome measures included nonunion, as defined by the need for re-operation to achieve fracture healing, malunion, varus collapse (defined as ten degrees of radiographic varus from index surgery), surgical complications, and postoperative medical complications, such as thromboembolic events, pneumonia, urinary infection, myocardial infarction, and death.

### Statistical analysis

Descriptive statistics were recorded for 264 patients, who completed their minimum twelve weeks follow-up appointment. All statistical analysis was performed using Stata 14 (StataCorp, College Station, TX). Given the relatively small number of events encountered for our main outcome measure (mechanical implant failure), no comparisons between the mechanical failure group and the non-mechanical failure group was performed. All continuous variables were tested for normal distribution. Normally distributed data was reported as means with standard deviation (SD). Not normally distributed data was reported as median with range.

## Results

Based on the Current Procedural Terminology (CPT) code 27245, a total of 756 patients were screened for participation in this study. One hundred seventy-three patients did not meet the inclusion criteria. The specific reasons for exclusion from the study were use of another fixation construct (*n* = 37), treatment with a different nailing system (*n* = 4), non-intertrochanteric fracture (*n* = 107), unrelated death (*n* = 16), and pathological fracture (*n* = 9). A total of 74 patients were screen failures including duplicates, coding errors, and non-identifiable patients. Thus, a total of 509 patients were enrolled in this study. Two hundred forty-five of the remaining 509 (48%) did not complete their minimum 12-week follow-up appointment. None of the 245 patients with incomplete 12-week follow-up data was found to have any signs of mechanical hardware failure at their latest follow-up appointment. The outcome data reported herein are based on 264 patients with complete follow-up data. The demographic and clinical data of these patients are listed in Table 1 and Table 2, respectively.

**Table 1:** Patient demographics

Age [years]	Mean 67.9 (Range 18–98)
Gender	
Female	144 (54.5%)
Male	120 (45.5%)
Diabetes mellitus	
No	202 (76.5%)
Yes	60 (22.7%)
Unknown	2 (0.8%)
Body mass index [kg/m^2^]	26.1 (Range 12.2 to 54.9)
Obesity	
Non-obese (BMI < 30.0 kg/m^2^)	205 (77.7%)
Obese (BMI ≥ 30.0 kg/m^2^)	59 (22.3%)
Injury mechanism	
Ground level fall	182 (68.9%)
Fall from height	30 (11.4%)
Motor vehicle collision	19 (7.2%)
Farm injury (attack by horse, bull, sheep)	4 (1.5%)
Bicycle accident	4 (1.5%)
Motorcycle collision	7 (2.7%)
Gunshot injury	2 (0.8%)
Motor vehicle versus pedestrian collision	3 (1.1%)
Crushed injury	2 (0.8%)
Other (golf cart, ATV, jet ski)	11 (4.2%)

**Table 2:** Clinical data

OTA/AO Fracture Classification	
A 1.1	45 (17%)
A 1.2	12 (4.5%)
A 1.3	6 (2.3%)
A 2.1	56 (21.2%)
A 2.2	48 (18.2%)
A 2.3	20 (7.6%)
A 3.1	20 (7.6%)
A 3.2	18 (6.8%)
A 3.3	39 (14.8%)
Time from orthopedic consultation to operative room [hours]	Mean 25.4 (Range: 1–456)
Length of hospital stay [days]	Mean 8.2 (Range: 1–55)
Length of follow up [weeks]	Mean 37 (Range: 12–186)
Operative time from skin incision [min]	Mean 95.87 (Range: 20–429)
Estimated blood loss [mL]	Mean 196.12 (Range: 5–1200)
Immediate postoperative neck-shaft angle [degrees]	Mean 127.38 (Range: 115–144)
Tip-apex distance	Mean (Range: 5.1–29.48)

The initial surgeries were performed between 2012 and 2016. 244 (92.4%) patients were operated at the two urban certified level-1 trauma centers and 20 patients (7.6%) were managed at the urban certified level-3 trauma center. All patients included in this study presented with an OTA/AO type 31-A fracture. The nail size and screw configuration was chosen based on the fracture pattern and the surgeon’s preference. Number of long nails (*n* = 202) used for implantation exceeded the number of short nails (*n* = 62). The nails of 217 patients (82.1%) included use of distal locking screws. Additionally, immediate postoperative imaging showed an average tip-apex distance of 16.2 mm [range: 5.1 mm - 29.5 mm] with a neck shaft angle of 127.4 degrees [range: 115 degrees - 144 degrees]. The mean estimated blood loss was 196.1 mL [range 5 mL – 1200 mL].

With regards to the primary outcome measure of mechanical hardware failure and screw cutout, we encountered a total of two screw cut outs among the 264 patients (0.75%). In one case of screw cut out, the implant was a long nail with one distal interlocking screw. The patient had an immediate post-operative neck-shaft angle of 136 degrees and a tip-apex distance of 6.8 mm. Upon follow up, non-union and varus collapse of 14 degrees was recorded. The patient underwent removal of hardware and total hip arthroplasty. The other screw cut out occurred in a patient with pre-existing avascular necrosis of the femoral head and, subsequently, underwent hardware removal and total hip arthroplasty at approximately 6 weeks postoperative. Regarding the nail construct, the immediate postoperative tip-apex distance was 13.3 mm with a neck shaft angle of 131 degrees. The implant was a short nail with one distal locking screw.

Other implant-related complications occurred in 19 cases (7.9%), which included broken distal screws (*n* = 9), distal screw loosening (*n* = 8), and loose lag screws (*n* = 2). Two other revision surgeries were performed for malrotation (*n* = 1) and malunion (*n* = 1). In addition, removal of symptomatic hardware was required in three patients (1.1%).

As for other secondary outcomes, two patients (0.75%) presented with delayed unions, both of whom were treated conservatively with either a bone stimulator and/or vitamin D supplementation. They subsequently went to bony union at their final follow-up. There were 30 Postoperative medical complications (11.4%), including acute renal injury (*n* = 5), urinary tract infections (*n* = 13), respiratory distress (*n* = 5), deep vein thrombosis (*n* = 1), pulmonary embolism (*n* = 1), pneumonia (*n* = 4), and myocardial infarctions (*n* = 1).

There was a total of 10 (3.8%) surgical postoperative complications, including four deep wound infections and six superficial wound complications. Three patients with deep wound infections were treated successfully with operative irrigation and debridements in addition to intravenous antibiotics while one patient had to undergo hardware removal. The six patients with superficial wound infections were successfully treated with oral antibiotics resulting in resolution of their symptoms. Data pertaining to complications is listed in Table 3.

**Table 3:** Complications

Mechanical hardware failure	
Screw cutout	2 (0.75%)
Broken distal screws	9 (3.4%)
Distal screw loosening	8 (3.0%)
Loose lag screws	2 (0.75%)
Delayed union	2 (0.75%)
Postoperative medical complications	
Acute renal injury	5 (1.9%)
Urinary tract infection	13 (4.9%)
Respiratory distress	5 (1.9%)
Deep vein thrombosis	1 (0.38%)
Pulmonary embolism	1 (0.38%)
Myocardial infarction	1 (0.38%)
Postoperative surgical complications	
Superficial wound infection	6 (2.3%)
Deep wound infection	4 (1.5%)
Revision surgery	
Malrotation	1 (0.38%)
Malunion	1 (0.38%)
Symptomatic hardware removal	3 (1.1%)

## Discussion

The incidence of hip fractures, such as intertrochanteric femoral fractures, is expected to double in the next 25 years due to the higher life expectancy of the population [18–21]. Because most of these patients will be elderly, operative management should consist of a stable construct performed in a timely manner to decrease both surgical and medical complications. Currently, several fixation techniques for these fractures consist of intramedullary nails, dynamic hip screws, or arthroplasty [7, 22]. The results of our retrospective study confirmed our hypothesis that this innovative two-screw cephalomedullary nail is a safe and reliable nailing system for the treatment of patients with intertrochanteric femoral fractures. In our series of 264 patients, we observed only two screw cut outs (0.75%).

Our mechanical failures of this study must be interpreted in the context of the patient demographics. One of the patients with screw cut out was elderly with pre-existing avascular necrosis of the femoral head while the other had a nonunion in the setting of acceptable tip-apex distance of 13.3 mm with a neck shaft angle of 131 degrees [13–15, 17]. Possible reasons for mechanical failure can be from surgical technique, position of lag screws, or tip apex distance. Hopp et al. [23] found mechanical failure to be correlated with the positioning of the lag screw but suggested that the surgeon’s techniques (closed reduction, positioning of lag screw), not implant configuration, is crucial in achieving successful outcome. Also, lag screw placement, specifically as it pertains to tip apex distance, has proven to be a meaningful calculation when predicting complications [13–15, 17]. Our study showed two possible preventable revision surgeries for malrotation and malunion, which can be the result of subpar surgical technique. These findings may represent the inherent risks of any intramedullary nail procedure, irrespective of the safety and effectiveness of the two-screw cephalomedullary nailing system itself. Our study did however have two loosening of lag screws, one of which required hardware removal. We also encountered nine broken distal screws and eight distal screw loosenings, whereby none of which were associated with any adverse outcomes. Overall, our findings are in line with other reports of this two-screw cephalomedullary nail found in the literature. Ruecker et al. [9] had two varus malalignment due to poor reduction in their study of 100 patients. Also, Jong-Won Kim et al. [24] encountered three complications requiring reoperation: two varus collapse with cut out and one case of periprosthetic fracture.

Several biomechanical and clinical data reported in the literature has shown favorable outcomes with the two-screw cephalomedullary nail construct [4, 6, 25]. Ruecker et al. [9] looked at 100 patients with stable and unstable intertrochanteric fractures treated with this two-screw cephalomedullary nail construct and found no loss of reduction, non-unions, or implant failures. Compared with the Synthes Proximal Femoral Nail Anti-rotation (PFNA), the two-screw cephalomedullary nail had significantly less complications, such as screw cut out, femoral shaft fracture distal to the tip of the nail, fracture collapse, and revisions [4, 25]. Also, the Stryker Gamma nail had a higher incidence of cut out and femoral shaft fracture compared to this two-screw cephalomedullary nail [6]. However, other studies reported equivalent outcomes. Jong-Won Kim et al. [24] found that only 45 out of their 100 patients (60.8%) with intertrochanteric femur fractures treated with the two-screw cephalomedullary nail recovered pre-fracture functional status. Also, Hopp et al. [23] found no differences between the two-screw nail and the Gamma3 nail in terms of mechanical failures and functional outcomes determined by the Harris Hip Score at 6 months postoperatively. Erez et al. [26] found rates of complications between the two-screw cephalomedullary nail and the Gamma nail to be similar.

Strengths of this study include its multicenter design. Also, our study looked at the clinical and radiographic outcomes in a large sample size with 264 patients treated with the two-screw cephalomedullary device. Limitations of our study include the retrospective design. Moreover, the patients were managed by different surgeons and according to different surgical and postoperative treatment protocols. Also, the study did not have a comparison group treated with a different nailing system. Finally, we encountered a loss of follow-up at 12 weeks of 48%, which is inherent for this patient population, but may potentially bias the study results. However, we would like to emphasize that none of the patients lost to follow-up had demonstrated signs of mechanical failure during their last postoperative visit. In addition, we carefully suggest that in case of any significant postoperative problems, a return to our trauma centers would have been likely for most of these patients.

Future studies on this cephalomedullary device should be conducted as multicenter trials given the low rate of mechanical failure, which necessitates a large sample size to capture and assess the effects of the implant. Given our results and the current status of the literature, we speculate that future studies will confirm low rates of hardware failure and screw cut out and will support this two-screw cephalomedullary nail as a safe and effective implant for the treatment of intertrochanteric fractures.

In conclusion, our multicenter study found that this two-screw cephalomedullary nail system had a low cut out and mechanical failure rate. Cephalomedullary nailing of intertrochanteric hip fractures using this improved system can be considered a safe and effective treatment method. Our findings confirmed that the two-screw cephalomedullary nail provides appropriate patient safety.

## Abbreviations

ATV: All-terrain vehicle
BMI: Body Mass Index
CPT: Current Procedural Terminology (CPT)
Ml: Milliliter
Mm: millimeter
OTA/AO: Orthopaedic Trauma Association/Arbeitsgemeinschaft für Osteosynthesefragen
